# Effect of ambient wind on the performance of alpine downhill skier

**DOI:** 10.1038/s41598-023-32107-4

**Published:** 2023-03-25

**Authors:** Bo Li, Peng Li, Yuanzhao Zhang, Kun Jia, Ping Hong

**Affiliations:** 1 School of Civil Engineering, Beijing Jiaotong University, Beijing, 100044 China; 2 Beijing’s Key Laboratory of Structural Wind Engineering and Urban Wind Environment, Beijing, 100044 China; 3 Beijing Sport University, Beijing, 100084 China

**Keywords:** Environmental social sciences, Applied mathematics

## Abstract

Alpine skiing, especially alpine downhill, is one of the most extreme winter sports in terms of high-speed and narrow winning margin, and its tracks are always located in mountainous areas with high altitudes and complex ambient wind fields, resulting in a significant impact of ambient wind on the performance and the final ranking of alpine downhill skiers. In the present study, a method based upon the combination of field measurements, wind tunnel tests and kinematic simulations was used to evaluate the effect of ambient wind on the performance of an alpine downhill skier. Considering the effect of ambient wind, a kinematic model of the alpine skier-ski system was established, and the equations of motion for straight gliding and turning were deduced. Then, the Chinese National Alpine Ski Center (CNASC) downhill track was taken as a case study to investigate the effect of ambient wind on the gliding time using the proposed combined evaluation method. Field measurements and wind tunnel tests were performed to identify five critical ambient wind directions of 270°, 292.5°, 315°, 337.5° and 360°. Moreover, the wind speeds and the wind directions for 16 different measurement points of the downhill track were also obtained. The results of the modelling analysis showed that the finish time increased by 19.75% for the ambient wind direction of 270°, whereas the finish time decreased by 1.29% for the ambient wind direction of 360°.

## Introduction

Alpine skiing is one of the most popular snow sports that perfectly combines speed and skill, is one of the official and signature Winter Olympic sports, and consists of five principle sub-projects: downhill (DH), slalom (SL), giant slalom (GS), super giant slalom (SG) and alpine combined (AC), of which the DH is the most extreme event in terms of competition speed with athletes reaching top speeds in excess of 140 km/h^1^. Additionally, the finishing time of the elite athletes in the race often varies by a mere hundredths of a second^2^, implying that any kinematic or kinetic factor may significantly influence the final ranking directly or indirectly, such as aerodynamic drag force, ski-snow interaction, equipment (for example skis, poles, and racing suits) and trajectory as well as the skiing techniques^3–5^. In particular, the alpine downhill tracks are always located in mountainous areas with high altitudes and complex ambient wind conditions. Due to these reasons, the influence of ambient wind on the gliding time of skiers cannot be ignored. Considering high speeds and narrow winning margins, athletes or coaches should have a clear idea of where time is being saved and lost so they can take appropriate measures and training to achieve the shortest racing time. Therefore, it is of significance to assess the performance throughout the whole competition, especially the consumption of time.

In recent years, several studies have been carried out to investigate the performance of alpine skiing events by combining wind tunnel experiments, field measurements^2,6–8^ and Computational Fluid Dynamics (CFD)^9–11^. Moreover, some researchers have focused on the motion of skier-ski system using modelling. Legotin and Rivlin^12^ developed a rod model of the skier-ski system to estimate various mechanisms of the loss of stable position in the process of performing a ski turn, including the lateral sliding and falling in the frontal plane. Nemec^13^ assumed that the skier behaves like an inverted pendulum and estimated the center of mass (COM) and the ski's trajectories. Based on the inverted pendulum model, a similar approach was proposed by Komissarov^14,15^. In the study of Cai and Yao^16^, the skier-ski system was modelled as a rigid body to investigate the physical dynamic characteristics and trajectory optimization. Besides, Chen and Qi^17^ developed a two-dimensional (2D) model to simulate skiing movements based on a multibody system, whereas a similar planar multibody simulation model was used to study the transversal vibrations during the schussing in the fall line over bumpy and rough ski slopes^18^. Oberegger^19^ established a 3D multibody skier model to simulate consecutive turns. It should be noted that, in all the aforementioned studies, aerodynamic drag is an important factor affecting the performance of alpine skiing events, which has been taken into account when developing the kinematics model. However, the current studies only consider the aerodynamic drag caused by the relative movement between the skier and the air, while ignoring the one caused by ambient wind, thus resulting in discrepancies between the results obtained from modelling analysis and the actual situations.

To date, investigations on the influence of ambient wind on the aerodynamic characteristics have extensively been performed in other sports such as cycling and ski jumping. For example, Fintelman^20,21^ performed an extensive numerical study and wind tunnel experiments to investigate the effect of crosswinds on the flow and aerodynamic forces of the bicycle system, and reported that different crosswind angles had significant effects on the vortex structures and aerodynamic force coefficients. Mannion et al.^22^ analyzed the crosswind aerodynamics of competitive hand-cycling under crosswind conditions using wind tunnel experiments and CFD simulations. As for the ski jumping, the ambient wind is closely associated with the fairness of the competition and the safety and stability of a ski jumper^23,24^. Jung^25^ investigated the influence of ambient wind on the jump length and flight technique optimization (including the angle of attack of the skis and the body-to-ski angle) of ski jumping during the flight phase. Hu and Liu^26^ performed CFD simulations to analyze the effects of different ambient wind conditions (including different horizontal, vertical and lateral ambient wind conditions) on the aerodynamic characteristics and stability during the flight in ski jumping. Although the aerodynamics of downhill skiing has been studied^10,11,27–29^, to the best of the author's knowledge, the detail analysis of the effects of ambient wind on alpine skiing has not yet been performed. Additionally, the ambient winds considered in the above studies are either ideal or fictitious, which are significantly different from the actual situation, thus limiting their practical applications. For a specific race, the wind environment of the track is relatively fixed during the competition. Therefore, the analysis of the performance of sports events under actual wind environment plays a key role in the intensive training of athletes and the decision-making of coaches.

In this study, to overcome the shortcomings presented in the previous review, a combined method consisting of field measurements, wind tunnel tests and kinematic modelling is established to evaluate the effect of ambient wind on the performance of alpine downhill skiers. The findings can provide guidance for athletes or coaches for preparing for various competitions and the selection of different strategies to optimize their performances. "Methodology" of the current paper introduces the analytical process of the combined method, which mainly includes the ambient wind assessment of the alpine downhill track through field measurements and scale terrain model wind tunnel tests. Considering the effect of ambient wind, the kinematic model for the alpine downhill skier-ski system is established; "Case study" takes the Chinese National Alpine Ski Center (CNASC) downhill track as an example and analyses the effect of ambient wind on the racing performance of alpine downhill skier using the combined method proposed in "Methodology". A brief summary of the findings from the present study is given in "Conclusions".

## Methodology

**Figure 1:**
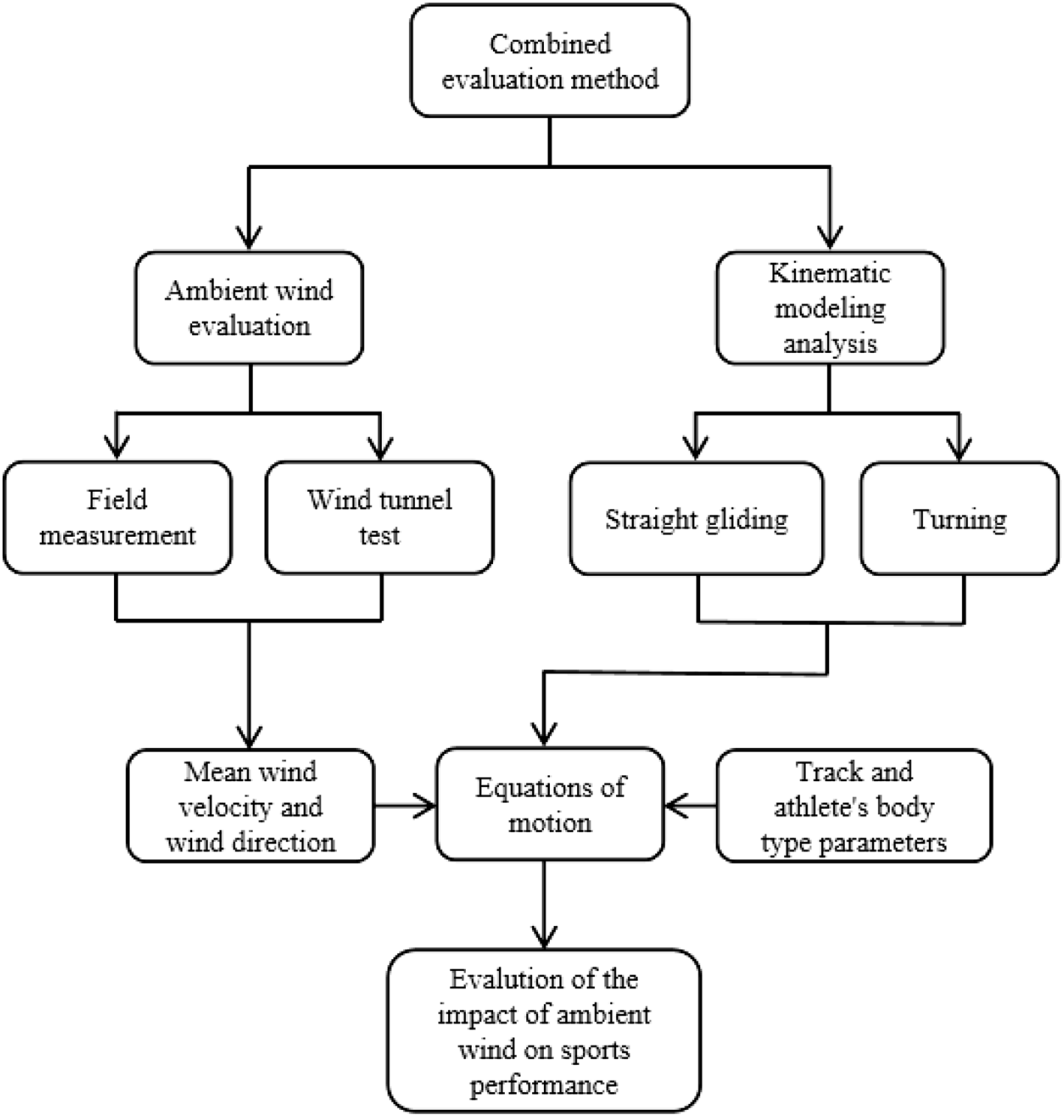
Analytical process of the combined evaluation method.

As shown in Fig. 1, the combined method consisted of two parts, the evaluation of ambient wind and the analysis of kinematic modelling. The mean wind speed and the wind direction of each measurement point were firstly obtained through field measurements and scale terrain model wind tunnel tests. Then, considering the effect of ambient wind, the model of motion of the alpine downhill skier-ski system was established. Finally, the mean wind speed and the wind direction were substituted into the established kinematic model as the parameters. Meanwhile, the track information and the athlete's body type parameters were substituted to solve the gliding time for each track interval using time iteration. Furthermore, the effect of ambient wind on the performance of athletes was evaluated.

### Evaluation of the ambient wind

#### Field measurements

Field measurements are the most direct and realistic way to grasp the characteristics of wind fields in complex terrain such as mountainous areas, and from which we can form a complete and long-term wind speed database. Moreover, field measurements are also the basis of subsequent wind tunnel tests and obtaining wind field information for the track. In the practical site-measurement process, information about the wind speed and the wind direction is usually measured and recorded continuously using wind measuring instrument such as a 3D ultrasonic anemometer. The collected wind data should first be preprocessed to ensure its validity, including the removal of all of the outliers and controlling the data continuity of time and space^30^.

The mean wind speed $\overline{U}$ and the mean wind direction $\theta$ are two of the most important factors in the performance of alpine downhill events that are affected by ambient winds. According to the site-measurement data collected by the ultrasonic anemometer, the $\overline{U}$ can be calculated by Eq. (1).

1 $$\overline{U} = \sqrt {\overline{{U_{x} }}^{2} + \overline{{U_{y} }}^{2} + \overline{{U_{z} }}^{2} } ,$$

where $\overline{{U_{x} }}$, $\overline{{U_{y} }}$ and $\overline{{U_{z} }}$ are the 10-min mean values of $U_{i} \left( t \right)$ (*i* = *x*, *y*, *z*) calculated from the wind speed time history along the north ($U_{x} \left( t \right)$), east ($U_{y} \left( t \right)$) and vertical ($U_{z} \left( t \right)$) directions, respectively.

As for the mean wind direction, it is assumed to be equivalent to the horizontal direction since the vertical angle is so small that it can be ignored. The wind direction $\theta$ is given by Eq. (2).

2 $$\theta = \arccos \left( {\frac{{\overline{{U_{x} }} }}{{\sqrt {\overline{{U_{x} }}^{2} + \overline{{U_{y} }}^{2} } }}} \right),$$

where the mean wind direction $\theta$ is calculated in degrees (°), and 0° and 90° represent the north and east directions, respectively.

#### Wind tunnel tests

Due to the complexity of the wind field in the mountainous areas, it is difficult to measure the wind speed in the field. In addition, if we want to obtain more comprehensive and accurate information on wind speed and wind direction, a large number of wind measuring instruments must be installed, which result in a significant increase in human and material costs. In order to overcome the shortcomings of field measurements, scale terrain model wind tunnel tests have become one of the most effective ways to investigate the characteristics of wind fields in complex environments such as mountainous areas.

In general, there are four main control systems that should be contained in the test equipment, including an income-flow wind speed control system, a wind direction control system, an automatic moving test frame system and a wind speed measurement system. More specifically, the income-flow wind speed is measured using a pitot tube. The scale terrain model is placed on a motor-driven turntable and the wind attack angle is controlled by controlling the angle of the turntable. The wind velocity is measured synchronously using the Turbulent Flow Instrumentation (TFI) Cobra Probe that is mounted on the automatic moving test frame system. Furthermore, spires and roughness elements are used to simulate a typical boundary layer wind flow corresponding to the actual conditions.

### Model of the motion of skier-ski system

**Figure 2:**
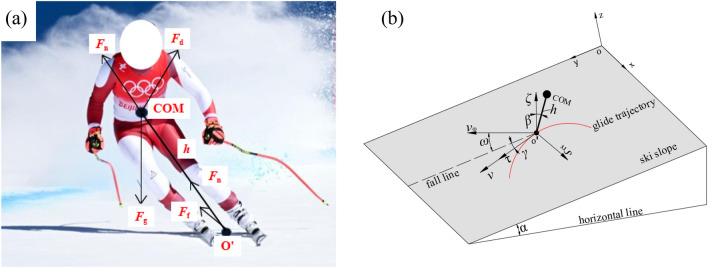
Skier-ski system and reference systems: (**a**) skier-ski system; (**b**) definition of coordinate system and direction angle.

In this sub-section, similar to the study of Cai and Yao^16^, the skier-ski system is treated as the COM connected to the supporting point (point *o'*) by a massless rod with constant length *h* (Fig. 2a). For simplicity, the whole mass of skier-ski system is equal to the sum of the mass of skis, poles and skier, and is concentrated at the COM. In addition, the effect of ambient wind on the motion of skier-ski system has also been considered.

#### Reference systems

Before establishing the equations of motion of the skier-ski system, it is necessary to give a detailed description of the coordinate systems and the definition of angle of direction involved in this paper.

As shown in Fig. 2b, the idealized case of ski slope with a constant gradient *α* was considered*.* Two reference systems were defined, including the global frame of reference *oxyz* and the local frame of reference *o'ξτζ*. In the *oxyz* coordinate system, axis *ox* and *oy* were along the horizontal and longitudinal directions of the ski slope, respectively. Moreover, the positive directions were from the west to the east and along the ski slope to the downhill direction, respectively. Additionally, the axis *oz* was perpendicular to the slope and oriented away from the slope in a positive direction. The supporting point *o'* was considered as the origin of *o'ξτζ* coordinate system. The axis *o'τ* and *o'ξ* were along the direction of motion speed *v* and the point to the center of curvature, respectively.

It should be noted that during the turning process of the alpine skiing, especially the DH event, the skier needs to control the left and right exchange of the center of gravity to realize the turn. In the model, the adjustment of the center of gravity is represented by the fact that the massless rod can rotate around the supporting point *o'*, resulting in an angle *β* between the massless rod and the normal of the ski slope (parallel to axis *o'ξ*). The angle *β* can reflects the degree of body tilt, which varies with the speed of movement. Additionally, the angle between the fall line and the gliding velocity vector *v* is defined as the track angle *γ*. Furthermore, the angle from fall line to ambient wind velocity vector *v*_*ω*_ is defined as *ω*. Meanwhile, both the *γ* and *ω* are positive in the anti-clockwise direction and negative in the clockwise, so *γ* is positive while *ω* is negative, as shown in Fig. 2b.

The angles *α*, *β*, and *γ* and the skier speed *v* satisfy Eq. (3)^31^.

3 $$\beta = \arctan \left( {\frac{{v^{2} }}{Rg\cos \alpha } + \tan \alpha \sin \gamma } \right).$$

#### Equations of motion

In order to derive comprehensible equations of motion of the skier-ski system, four assumptions are used to simplify the derivation of these equations: (1) during skiing (both straight gliding and turning), there is no skidding or take-off for the whole system, which means that the skis are always in full contact with the ski slope surface; (2) the skier-ski system will generate lateral forces under the action of ambient wind, indicating that the athlete has a tendency to drift (especially when gliding straight along the fall line). Due to this reason, it is assumed that the athlete can maintain the state of motion through the action of the joint such as the knees; (3) during turning, edging angle (the angle between the ski slope and the sliding surface of the ski) is considered to be numerically equal to the body inclination *β*; (4) for simplicity, the difference of trajectory between the COM and the ski is disregarded and both are considered to be consistent throughout the skiing.

As is known, the performance time of an alpine skier is a function of skier’s speed and trajectory, both of which are determined by the balance of the external forces acting on the skier-ski system. On the ski slope, the forces generated at the whole skier-ski system are shown in Fig. 2a, and include the gravitational force *F*_g_, the reactionary force of the ski *F*_n_, the snow friction force *F*_f_ and the aerodynamic drag force *F*_d_. According to D’Alembert's principle, the whole system satisfies the following equilibrium condition (Eq. 4).

4 $$m\vec{a} + \vec{F}_{{\text{g}}} + \vec{F}_{{\text{n}}} + \vec{F}_{{\text{f}}} + \vec{F}_{{\text{d}}} = \vec{0},$$

where the product of the whole mass of skier-ski system (m) and the acceleration of COM (a) represents the inertial force of the system.

**Figure 3:**
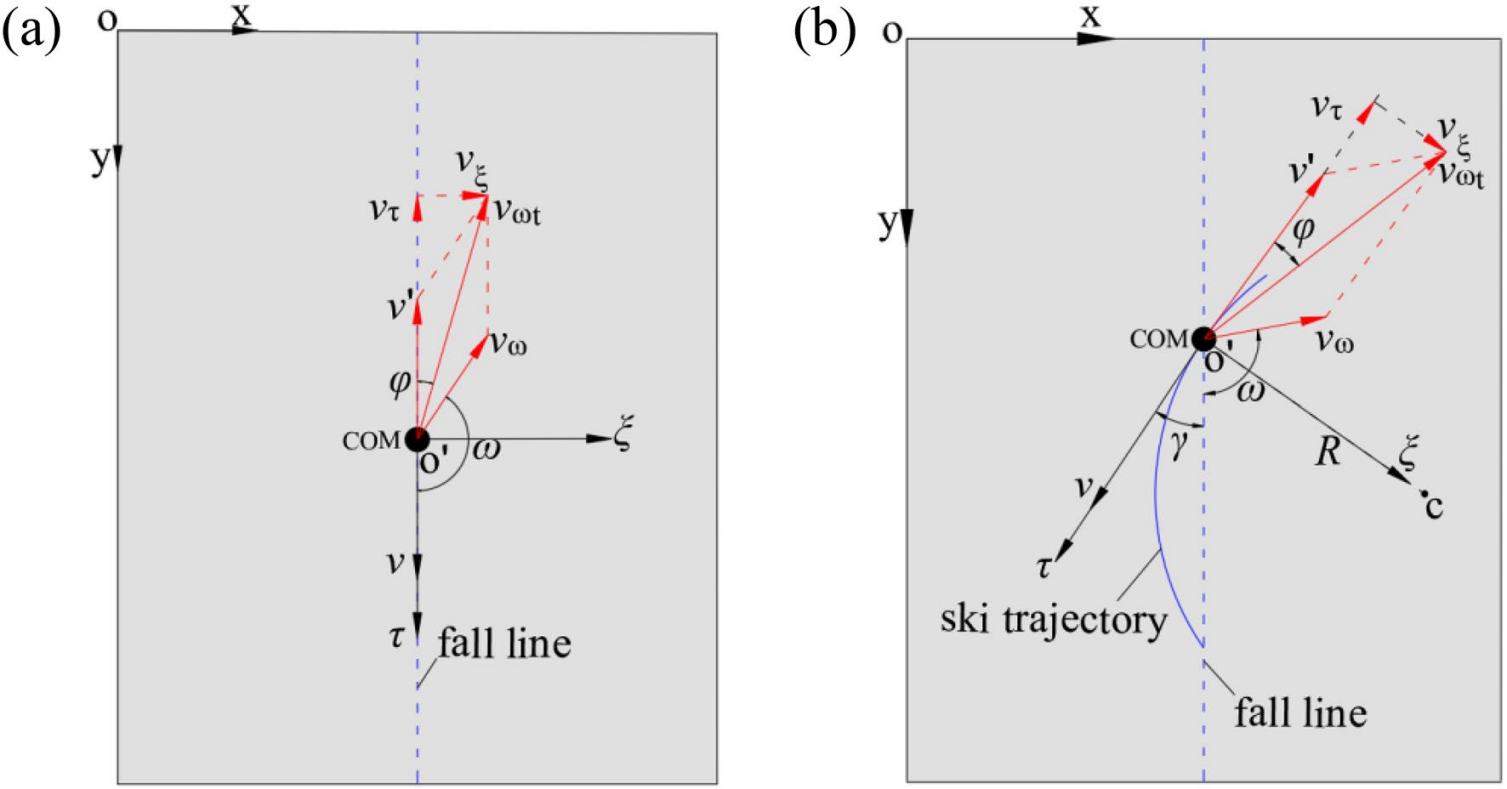
Schematic of the decomposition of ambient wind: (**a**) straight gliding; (**b**) turning.

The snow friction force *F*_f_ is parallel and in the opposite direction to skier’s velocity vector ***v*** and its magnitude is related to the reaction force of the ski *F*_n_^31^. The expression of *F*_f_ is denoted by Eq. (5).

5 $$F_{{\text{f}}} = \mu F_{{\text{n}}} ,$$

where *μ* is the friction coefficient. As for the reaction force of the ski *F*_n_, the direction is perpendicular to the ski and along the massless rod, so its magnitude is given by Eq. (6).

6 $$F_{{\text{n}}} = \left\{ \begin{gathered} mg\cos \alpha \quad \;\quad {\text{for}}\;{\text{straight}}\;{\text{gliding;}} \hfill \\ \frac{mg\cos \alpha }{{\cos \beta }}\quad \quad \,{\text{for}}\;{\text{turning,}} \hfill \\ \end{gathered} \right.$$

where *α* and *β* are the angle of the ski slope and the edging angle of skis, respectively, and g is the gravitational acceleration. The aerodynamic drag force *F*_d_ is calculated using Eq. (7).

7 $$F_{{\text{d}}} = \frac{1}{2}C_{d} \rho Av^{2} ,$$

where *C*_*d*_ is the drag coefficient, *ρ* is the air density, *A* is the cross-sectional area of the skier normal to the direction of wind, and *v* is the kinematic velocity. In particular, *C*_*d*_*A* is often obtained from the wind tunnel tests in the form of a product. However, due to the consideration of the effect of ambient wind, the aerodynamic drag force *F*_d_ is no longer parallel to the skier velocity vector ***v***. On the contrary, it is offset by a certain angle. On the ski slope, the effect of ambient wind on the COM is analyzed (Fig. 3). According to the vector analysis, Eqs. (8) and (9) are obtained.

8 $$\vec{v}_{\omega t} = \vec{v^{\prime}} + \vec{v}_{\omega } ,$$

9 $$\vec{v^{\prime}} = - \vec{v},$$

where $\vec{v}_{\omega t}$,$\vec{v}_{\omega }$ and $\vec{v}$ are the resultant wind velocity vector, ambient wind velocity vector and skier velocity vector, respectively. Here, the angle between $\vec{v}_{\omega t}$ and $\vec{v}$ is defined as *φ*. Then, the ambient wind velocity vector $\vec{v}_{\omega }$ is decomposed along the axis *o'τ* and *o'ξ*, Eqs. (10) and (11) are obtained.

10 $$v_{\tau } = \left\{ \begin{gathered} v_{\omega } \cdot \cos \omega + v\quad \;\quad \quad \quad {\text{for}}\;{\text{straight}}\;{\text{gliding;}} \hfill \\ v_{\omega } \cdot \cos (\omega - \gamma ) + v\quad \quad \,{\text{for}}\;{\text{turning,}} \hfill \\ \end{gathered} \right.$$

11 $$v_{\xi } = \left\{ \begin{gathered} v_{\omega } \cdot {\text{sin}}\omega \quad \;\quad \quad \quad {\text{for}}\;{\text{straight}}\;{\text{gliding;}} \hfill \\ v_{\omega } \cdot \sin (\omega - \gamma )\quad \quad \,{\text{for}}\;{\text{turning}}{.} \hfill \\ \end{gathered} \right.$$

Then, *φ*, $F_{{\text{d}}}$, $F_{{{\text{d}}\tau }}$ and $F_{{{\text{d}}\xi }}$ are calculated by Eqs. (12) and (13).

12 $$\varphi = \arctan \left( {\frac{{v_{\xi } }}{{v_{\tau } }}} \right) = \arctan \left( {\frac{{v_{\omega } {\text{sin}}\omega }}{{v_{\omega } \cos \omega + v}}} \right),$$

13 $$\left\{ \begin{gathered} F_{{\text{d}}} = \frac{1}{2}C_{d} \rho A_{l} v_{{\omega {\text{t}}}}^{2} \hfill \\ F_{{{\text{d}}\tau }} = F_{{\text{d}}} \cdot \cos \varphi \hfill \\ F_{{{\text{d}}\xi }} = F_{{\text{d}}} \cdot \sin \varphi , \hfill \\ \end{gathered} \right.$$

where *A*_*l*_ is the cross-sectional area of the skier normal to the direction of the resultant wind velocity.

**Figure 4:**
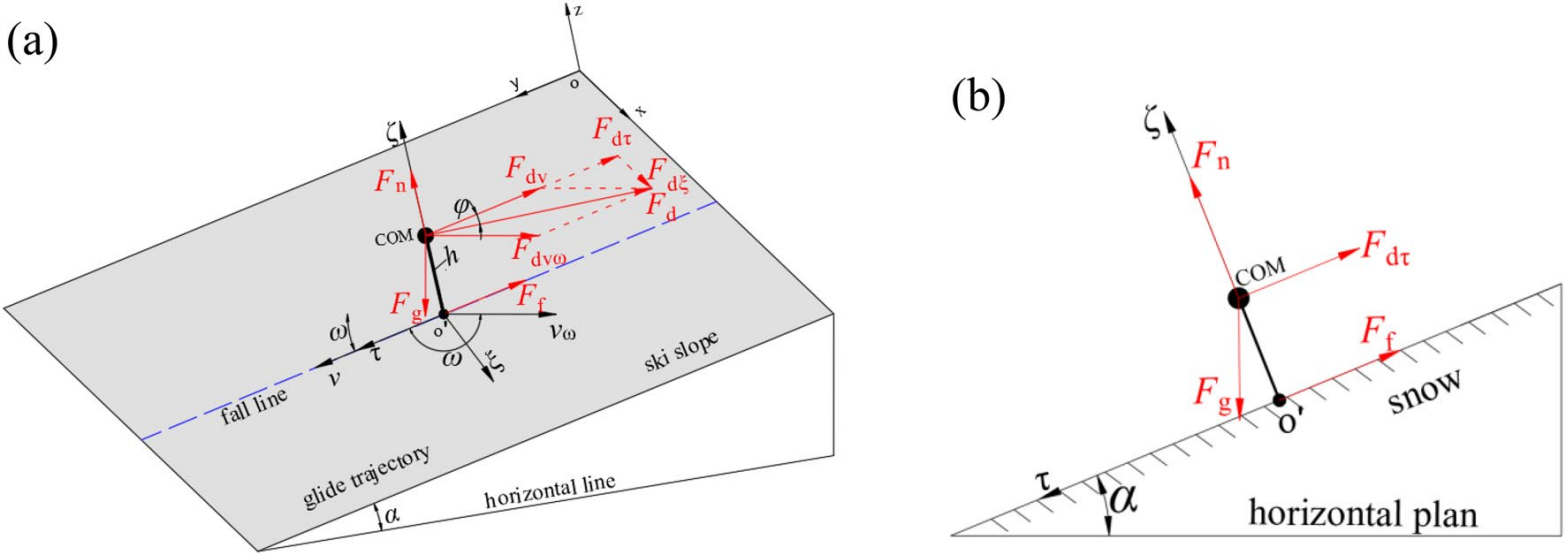
Schematic of the force decomposition during straight gliding: (**a**) forces at each point of action of the model; (**b**) forces at COM described in the local frame of reference *o'ξτζ*.

In the case of straight gliding down the fall line on the ski slope, the force analysis on the skier-ski system is performed (as shown in Fig. 4). Projecting all the forces experienced by the system onto the axis *o'τ*, Eq. (14) is obtained.

14 $$m\frac{dv}{{dt}} = F_{g} \sin \alpha - F_{f} - F_{d\tau } .$$

Substituting Eqs. (3)–(5), and (10)–(11) into Eq. (12), the kinematic equation of the system along the direction of skier velocity can be expressed as Eq. (15).

15 $$\frac{dv}{{dt}} = g(\sin \alpha - \mu \cos \alpha ) - \frac{{\rho C_{d} A_{l} \cos \varphi }}{2m}(v_{\omega }^{2} + v^{2} - 2v_{\omega } \cdot v \cdot \cos \omega ),$$

where *φ* is calculated by Eq. (12).

**Figure 5:**
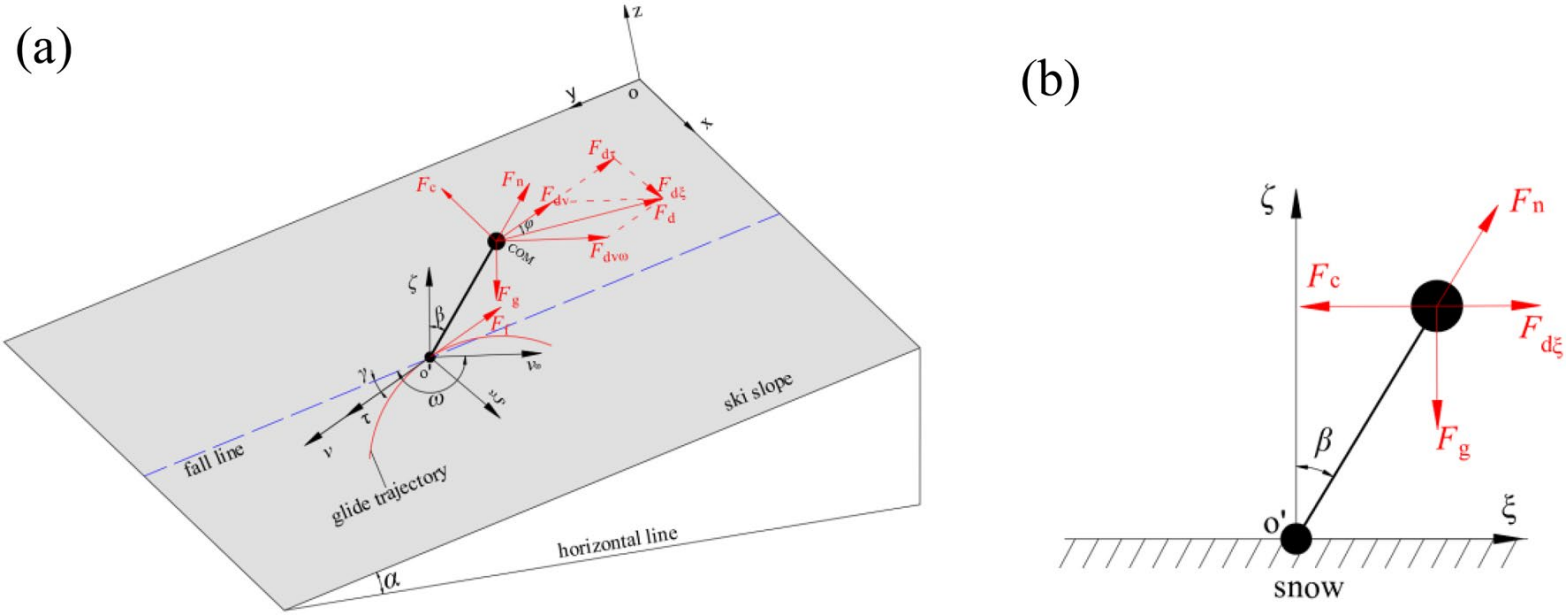
Schematic of the force decomposition during turning: (**a**) forces at each point of action of the model; (**b**) forces at COM described in the local frame of reference *o'ξτζ*.

In the case of turning on the ski slope, the forces acting on the skier-ski system are similar with those in the straight gliding down the fall line in the direction of skier velocity, while they are different in the direction perpendicular to the skier movement due to the presence of centrifugal force (as shown in Fig. 5). Similar to Eq. (15), the kinematic equation of the system along the direction of skier velocity is expressed by Eq. (16).

16 $$\frac{dv}{{dt}} = g\left( {\sin \alpha \cdot {\text{sin}}\beta - \frac{\mu \cos \alpha }{{\cos \beta }}} \right) - \frac{{\rho C_{d} A_{l} \cos \varphi }}{2m}\left[ {v_{\omega }^{2} + v^{2} - 2v_{\omega } \cdot v \cdot \cos \left( {\omega - \gamma } \right)} \right].$$

When projecting the forces acting on the COM onto the axis *o'ξ*, the influence of ambient wind on the state of motion of COM can be divided into two categories due to the uncertainty of the direction of ambient wind. On the one hand, when the component of the resultant aerodynamic drag force along the direction perpendicular to the movement velocity is towards the center of curvature, this component can provide centripetal force to the skier, which increases his or her speed during turning. On the other hand, when the component of the resultant aerodynamic drag force along the direction perpendicular to the movement velocity is away from the center of curvature, this component can provide centrifugal force to the skier and reduce his or her turning speed. According to the force analysis, Eq. (17) can be obtained.

17 $$F_{c} = F_{g} \sin \alpha \cdot \sin \gamma + F_{n} \sin \beta \pm F_{d\xi } ,$$

where *F*_*c*_ is given by Eq. (18).

18 $$F_{c} = mv\omega \quad \quad {\text{where}}\quad \omega = \frac{d\gamma }{{dt}}.$$

Substituting Eqs. (5)–(7), (10)–(13) and (18) into Eq. (17), Eq. (19) is obtained.

19 $$\frac{d\gamma }{{dt}} = \frac{1}{v}\left[ \begin{gathered} g\cos \alpha \cdot {\text{tan}}\beta - g\sin \alpha \cdot \sin \gamma \pm \frac{{\rho C_{d} A_{l} \sin \varphi }}{2m} \hfill \\ \left( {v_{\omega }^{2} + v^{2} - 2v_{\omega } \cdot v \cdot \cos \left( {\omega - \gamma } \right)} \right) \hfill \\ \end{gathered} \right],$$

where the selection of positive or negative signs corresponds to the aforementioned classification of the influence of ambient wind on the state of the motion of COM.

**Figure 6:**
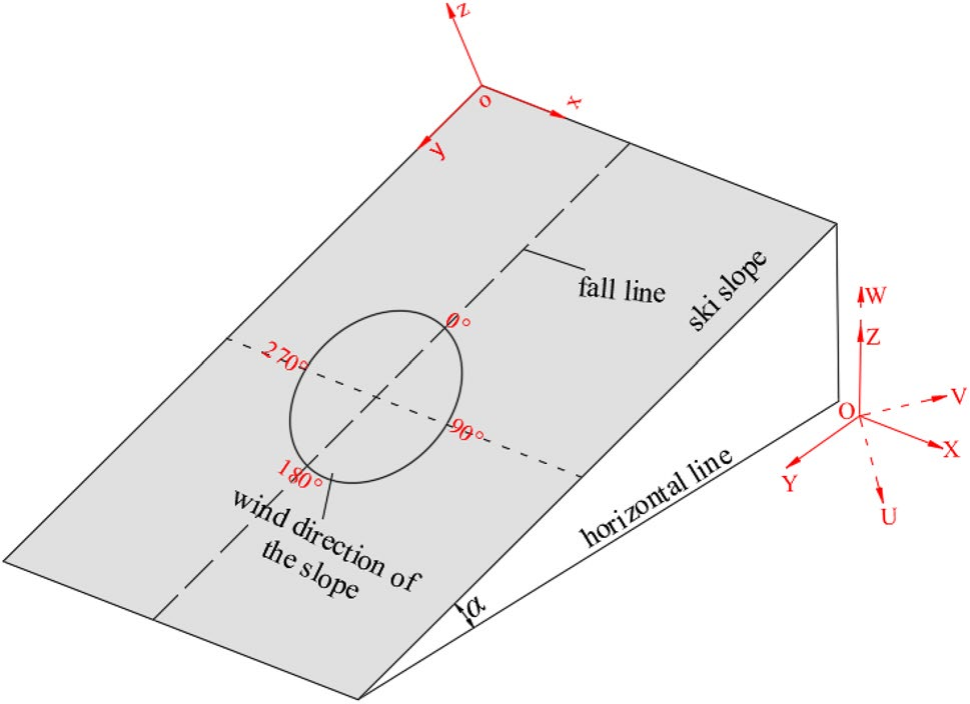
Schematic of the wind transformation.

It should be noted that the wind speed and the direction data of each measurement point obtained by wind tunnel test are corresponding to that in the wind axis coordinate system. When the state of the motion of the alpine skier is being analyzed, the ambient wind conditions in the plane of the gliding slope need to be taken into account. Therefore, it is necessary to convert the wind speed of each measurement point obtained from the wind tunnel test into the wind speed of the gliding slope. The transition coordinate system for data conversion is defined as the ground coordinate system *OXYZ*, whereas *X-*axis and *Y-*axis lie in the horizontal plane. The positive direction of *X-*axis is from west to east, while the *Z-*axis is perpendicular to the horizontal plane with the positive direction being the upward direction. The wind axis coordinate system *OUVW* is also defined, and the direction of approaching flow in the wind tunnel is specified as the positive direction of the *U-*axis*.* The schematic of the wind transformation is depicted in Fig. 6.

Firstly, the ambient wind speed and the direction data in the wind axis coordinate system are converted to the ground coordinate system, and the coordinate transformation is given by Eq. (20).

20 $$\left\{ \begin{gathered} X = u \cdot \cos \left( {\omega^{\prime} - 270^{ \circ } } \right) + v \cdot \sin \left( {\omega^{\prime} - 270^{ \circ } } \right) \hfill \\ Y = u \cdot \sin \left( {\omega^{\prime} - 270^{ \circ } } \right) - v \cdot \cos \left( {\omega^{\prime} - 270^{ \circ } } \right) \hfill \\ Z = w, \hfill \\ \end{gathered} \right.$$

where *u*, *v* and *w* are the three components of the measurement wind speed along the three axis of wind axis coordinate system, and *ω*' is the angle between the *U-*axis and the *X-*axis.

Then, the wind speed and the direction data from the ground coordinate system are transformed to the slope coordinate system *oxyz* (the same as the global frame of reference defined in "Reference systems"), where the ground coordinate system coincides with the slope coordinate system on the *X*-axis, but not with the *Y*-axis and *Z*-axis. Moreover, the angle between the two coordinate systems is numerically equal to the gradient of the ski slope. The coordinate transformation is given by Eq. (21).

21 $$\left\{ \begin{gathered} x = X \hfill \\ y = Y \cdot \cos \alpha - Z \cdot \sin \alpha \hfill \\ z = Y \cdot \sin \alpha + Z \cdot \cos \alpha . \hfill \\ \end{gathered} \right.$$

#### Estimation of the drag area C_d_A

It is well known that, when the ambient wind is not taken into account, the aerodynamic drag of the athlete is parallel to the movement velocity vector, and the two parameters of drag coefficient *C*_*d*_ and cross-sectional area of skier *A* (as given in Eq. (7)) remain constant. However, when considering the influence of ambient wind on the performance of skier, the aerodynamic drag force is no longer parallel to the skier velocity vector, but corresponds to the resultant velocity vector of the motion velocity vector. Moreover, the ambient wind velocity vector has a certain angle with the skier velocity vector. Therefore the drag coefficient *C*_*d*_ and the cross-sectional area of skier *A* will not remain constant. In order to better understand the influence of ambient wind on the performance of the skier, it is essential to accurately calculate the aerodynamic drag. In other words, it is necessary to investigate the variation trend of drag coefficient *C*_*d*_ and cross-sectional area *A* with ambient wind speed and its direction. In particular, a limitation with many wind tunnel systems is the inability to measure the cross-sectional area *A* of an irregular moving object. Therefore, *C*_*d*_*A* is often given by wind tunnel tests in the form of a product.

**Figure 7:**
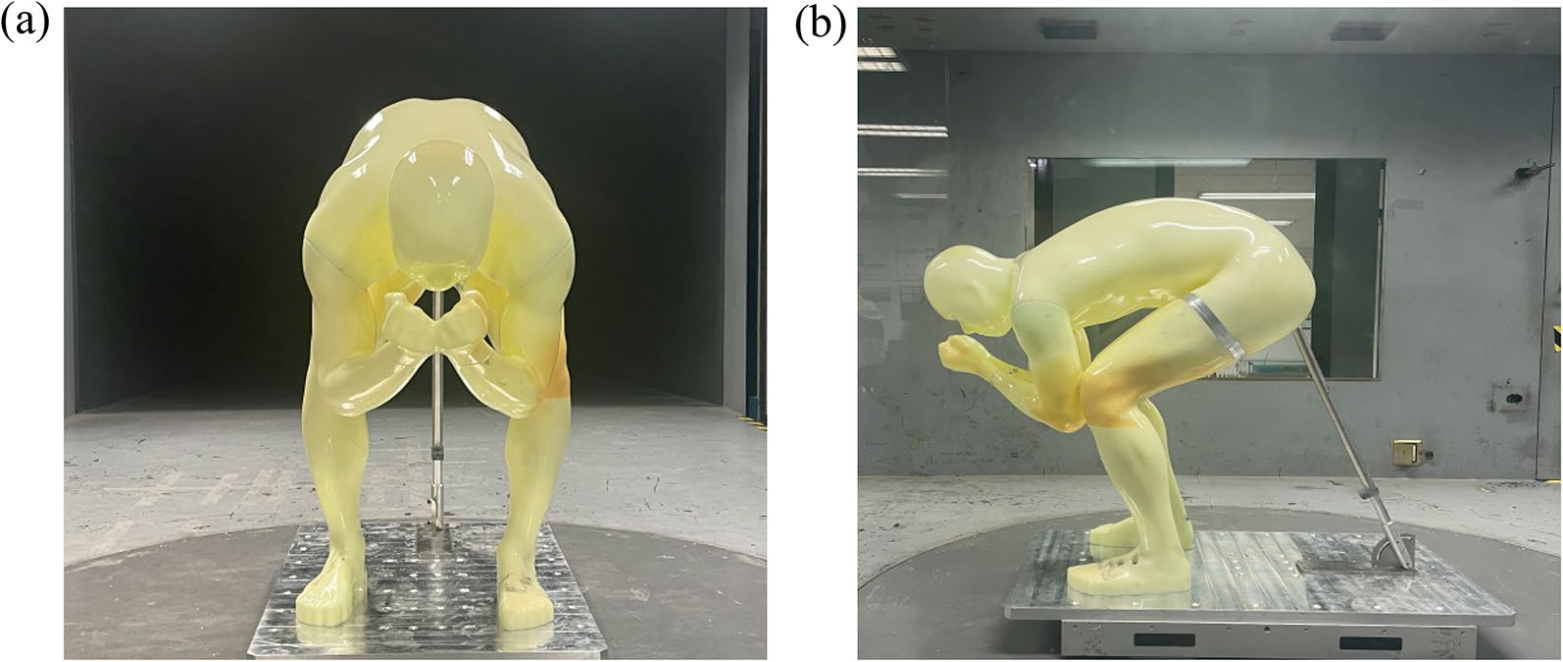
Full-scale mannequin of an alpine downhill skier for wind tunnel tests: (**a**) front view; (**b**) side view.

In order to better understand the variation pattern of *C*_*d*_*A* with wind speed and wind direction, a full-scale mannequin of an alpine skier (height: 1.80 m; body mass: 72 kg) was fabricated (as shown in Fig. 7) and a force platform test was carried out in the high-speed testing section of the Boundary Layer Wind Tunnel in Beijing Jiaotong University, China. The dimensions of the high-speed testing section had the width, height and length of 3.0 m, 2.5 m and 15.0 m, respectively. The maximum wind speed was 45 m/s and the turbulence intensity of even flow was less than 0.5%, indicating that the flow quality was excellent. Six-component force platform was used to measure the overall aerodynamic force on the mannequin. The sampling frequency and the sampling length were 1500 Hz and 15 s, respectively. The total aerodynamic force in the wind tunnel was calculated from the average unsteady drag force over a period of 15 s.

In the present study, the wind attack angle is defined as the angle between the direction of the approaching flow and the orientation of the mannequin, which is set from 0° to 21° in increments of 3°. Furthermore, the wind speed is set at nine different values from 10 to 26 m/s in the increments of 2 m/s. Data acquisition was repeated twice for each testing condition to minimize the measurement errors. Moreover, a tail support device was used to eliminate the negative effect of the model’s vibration on the test results.

**Figure 8:**
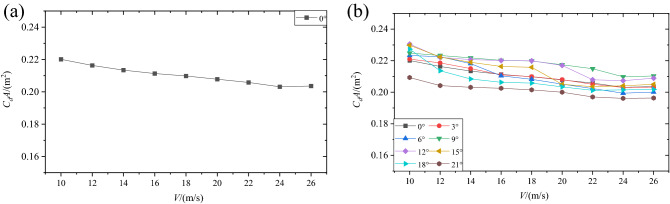
Variation of *C*_*d*_*A* with wind velocity: (**a**) for the wind attack angle of 0°; (**b**) under eight different wind attack angles.

Figure 8 demonstrates the variation of *C*_*d*_*A* with wind speed at different wind attack angles. It can be observed clearly that the variation trend of *C*_*d*_*A* with wind speed under different wind attack angles was almost the same, and the magnitude of *C*_*d*_*A* changed within a small range (from 0.2250 ± 0.05 m^2^ at 10 m/s to 0.2050 ± 0.05 m^2^ at 26 m/s) at different wind attack angles. On the one hand, at a certain wind attack angle (for example, for 0°, as shown in Fig. 8a), when the wind speed was low, the drag area *C*_*d*_*A* decreased with the increase of wind speed, while it remained almost constant for the high wind speed (over 24 m/s). Similar results were reported in Elfmark’s^32^ research. On the other hand, when considering the impact of wind angle of attack on *C*_*d*_*A*, it can be seen from Fig. 8b that, when the wind angle of attack was within the range of 0°–15°, the *C*_*d*_*A* of the mannequin was approximately equal at all wind speeds. However, when the wind angle of attack reached 21°, the *C*_*d*_*A* was approximately equal to other cases at higher wind speeds (over 24 m/s) and the difference was relatively large at lower wind speeds. The findings herein are in good agreement with previous studies. For example, *C*_*d*_*A* was calculated to be 0.19 m^2^ for a fully tucked position at 25 m/s^10^ and 0.23 ± 0.03 m^2^ for a tucked position at 22.2 m/s wind speed^33^. The slight difference was due to the slightly different posture and human parameters of the prototype of the mannequin.

## Case study

In order to better illustrate the evaluation method established in "Methodology", the CNASC downhill track was taken as an example to describe the evaluation process in detail. The alpine downhill track is one of the four main tracks of the CNASC. The track is 3085 m in length, starting at an elevation of 2190 m and ending at an elevation of 1345 m, thus making it the longest snow track with the largest difference in height in China. In particular, due to the high altitude of the track area (about 800 m down from the top of the mountain in the whole track) and the reason that there is no shelter, the wind speed is significantly higher than those in other areas. Meanwhile, because it is in the initial stage of slip and there is a large turning interval, the ambient wind has a significant impact on the state of motion of the slide. Therefore, only this area was considered in the present study. In this section, the ambient wind characteristics of the CNASC’s downhill track were firstly evaluated through meteorological data analysis and scale terrain model wind tunnel tests. In addition, the effect of ambient wind on the gliding time of alpine downhill skier was investigated.

### Evaluation of the ambient wind of CNASC’s downhill track

#### Field measurements

**Figure 9:**
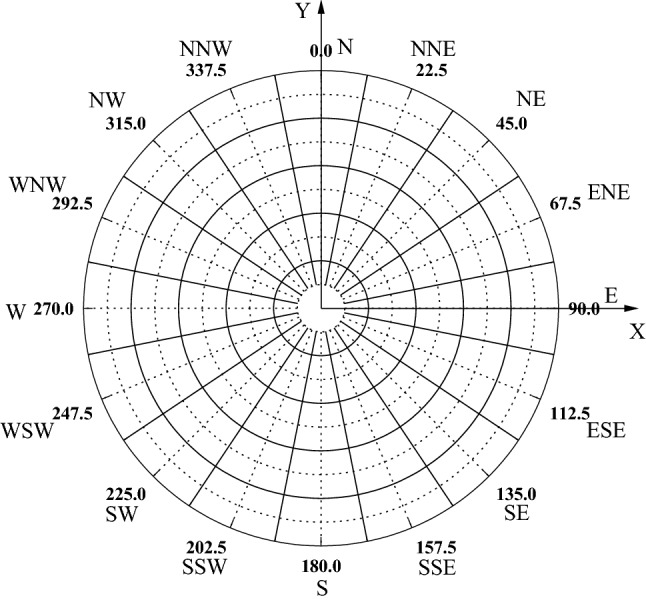
Definition of ambient wind angle.

Since the Beijing Winter Olympics of February 2022, the authors focused on analyzing the wind speed data of the meteorological station in February 2019. The meteorological station, with an elevation of 2194 m, was located at the mountaintop of the downhill track in CNASC. According to the actual wind speed measurement records provided by the Beijing Meteorological Bureau (China), the number of days of 10-min mean wind speed in February was statistically analyzed. Additionally, the relationship between the wind direction and the 10-min mean wind speed was analyzed. In order to describe the direction of the ambient wind, according to the wind speed observation specification of the meteorological department, the wind direction angle *ω*' was divided into 16 azimuths, as shown in Fig. 9.

The number of days with different ambient wind speeds in February, 2019 is presented in Table 1. According to the statistical results, the Meteorological Station S1 had 10.6 days in February 2019, with a 10-min mean wind speed of over 10 m/s, and 3.9 days with 10-min mean wind speed of more than 15 m/s. These values correspond to the probabilities of 37.86% and 13.93%, respectively, indicating that the effects of ambient wind to the performance of alpine skier cannot be ignored during February.

**Table 1:** Statistical data of days with different wind speeds at S1.

10-min mean wind speed (m/s)	Number	Probability (%)	10-min mean wind speed (m/s)	Number	Probability (%)
0–2.5	1.6	5.71	15.0–17.5	2.2	7.86
2.5–5.0	5.5	19.64	17.5–20.0	1.4	5.00
5.0–7.5	6.3	22.50	20.2–22.5	0.3	1.07
7.5–10.0	4.0	14.29	22.5–25.0	0	0
10.0–12.5	3.7	13.21	25.0–27.5	0	0
12.5–15.0	3.0	10.72	27.5–30.0	0	0

**Figure 10:**
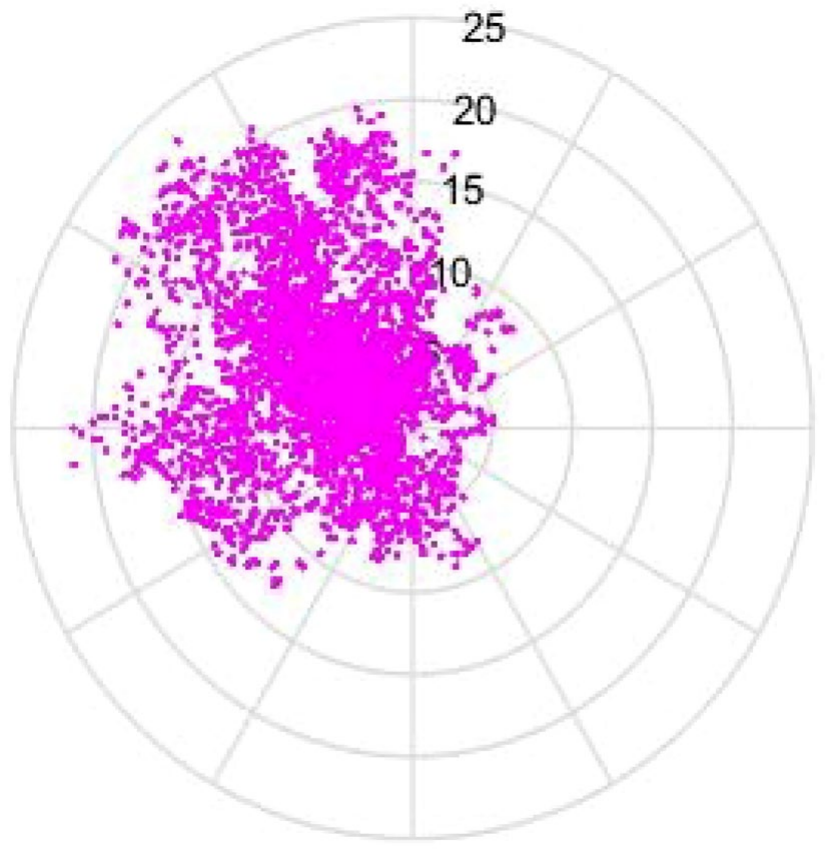
Velocity and direction of 10-min mean wind speed (unit: m/s).

Figure 10 shows the velocity and direction of 10-min mean wind speed in February 2019, from which it is clear that the ambient wind occurred mostly within the range of 270°–360°. Therefore, it was important to statistically analyze the ambient wind data within this range. The number, frequency, median and maximum of ambient wind speed are presented in Table 2. In general, the ambient winds occurred frequently within the range of 270°–360°. The maximum wind speeds were higher than those in the other directions, reaching around 20 m/s. The wind direction with the highest frequency of ambient winds was found in the direction of 315°, while the maximum wind speed also occurred in the same direction. In terms of median, the maximum and minimum median values of ambient wind occurred in the directions of 337.5° and 292.5°, respectively.

**Table 2:** Statistics of different ambient wind directions.

Direction (°)	270°/W	292.5°/WNW	315°/NW	337.5°/NNW	360°/N
Number	547	722	1007	578	335
Frequency (%)	13.57	17.91	24.98	14.34	8.31
Median (m/s)	7.3	6.9	9.6	12	7.8
Maximum	21.3	21.7	22.8	20.8	19.7

#### Scale terrain model wind tunnel tests

**Figure 11:**
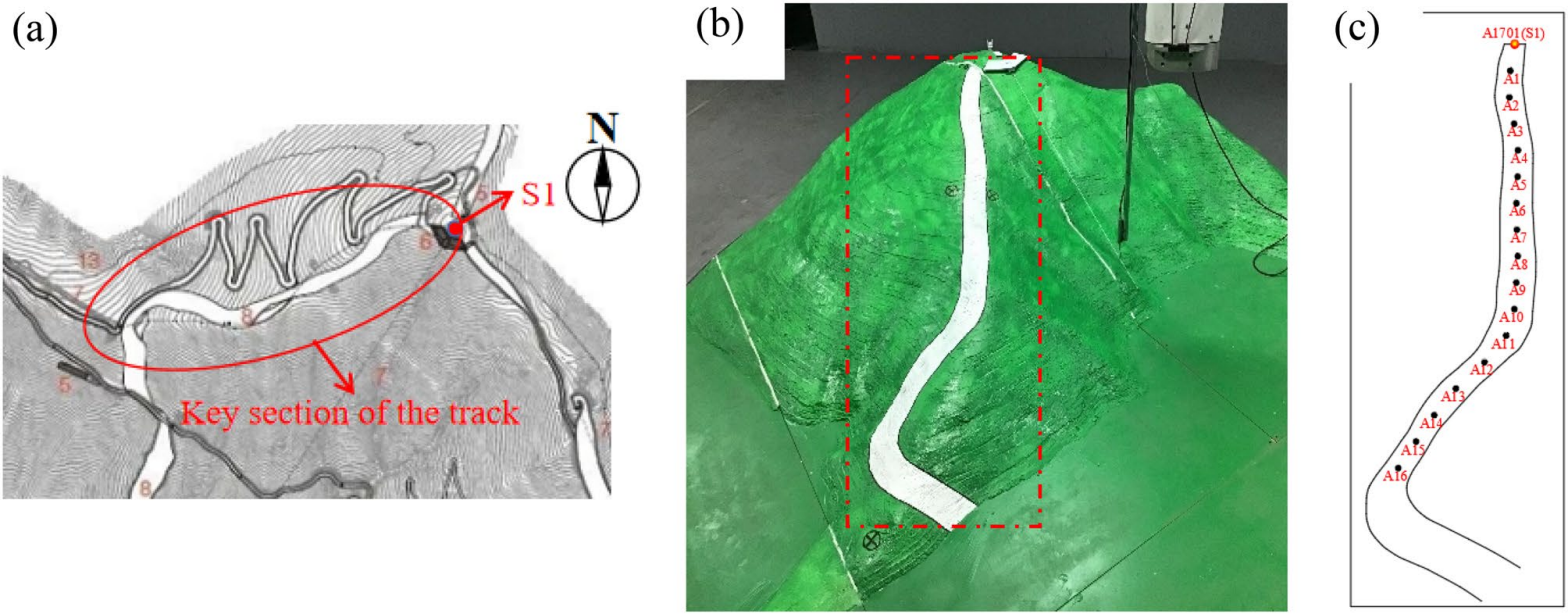
Terrain model and distribution of measurement points: (**a**) the plan of the alpine downhill track in CNASC; (**b**) terrain model of alpine downhill track; (**c**) distribution of measurement points.

Wind tunnel test of scale terrain model is an important method to obtain large-scale mountain wind field data. According to the topographic map of the CNASC competition area, a 1:500 scaled terrain model of the local area of the top of the mountain was constructed, and covered the topography of the local area of the mountain with a diameter of about 1.5 km. Moreover, the model completely covered the key section of the alpine downhill track in CNASC (see Fig. 11a). In order to avoid the abrupt change of the model edge affecting the test results, the transition treatment similar to the deflector was carried out at the abrupt change of the model edge. The terrain model is shown in Fig. 11b. Sixteen wind speed measurement points, labelled A1–A16, were distributed along the track (see Fig. 11c). The horizontal distance of each location was about 50 m. The location of meteorological station A1701 (also called S1) is marked in Fig. 6b. The elevations of each measurement point are presented in Table 3.

**Table 3:** Parameters of measurement points.

Measurement point	Elevation/m	Section	Type	Distance/m	Gradient *α*/°
A1	2180	–	–	–	–
A2	2170	A1A2	Straight	50	25.7
A3	2150	A2A3	Curved	61.1	14.0
A4	2130	A3A4	Curved	61.1	10.4
A5	2110	A4A5	Straight	50	15.7
A6	2100	A5A6	Curved	61.1	16.6
A7	2095	A6A7	Curved	61.1	15.2
A8	2080	A7A8	Straight	50	15.5
A9	2065	A8A9	Straight	50	17.4
A10	2060	A9A10	Straight	50	10.4
A11	2050	A10A11	Straight	50	1.1
A12	2040	A11A12	Straight	50	15.5
A13	2020	A12A13	Curved	61.1	18.0
A14	2005	A13A14	Curved	61.1	16.0
A15	1990	A14A15	Curved	61.1	16.2
A16	1975	A15A16	Curved	61.1	20.0
		–	–	–	–

In this study, wind tunnel tests were carried out in the low-speed section of the Closed-circuit Wind Tunnel in Beijing Jiaotong University (China). The test section was 5.2 m wide, 2.5 m high and 14.0 m long. The maximum test wind speed was 18 m/s, which meets the test requirements. The wind velocities of each location of wind speed measurement point and the location of Meteorological Station S1 were measured using a TFI Cobra Probe with a total length of 180 mm. The prob length was 30 mm with a 4-hole head 2.6 mm in diameter. The three-dimensional shifting measuring frame, with an error range of ± 0.1 mm, was used to precisely locate the position of the TFI cobra probe. The wind speed of approaching flow was 12 m/s. The sampling frequency and the sampling length were 1500 Hz and 36 s, respectively.

In order to obtain the velocity of ambient wind at each measurement point, the experimental ambient wind velocity was firstly normalized using the experimental wind velocity of Meteorological Station S1. The wind speed ratio *R*_w_ between the two was obtained. The dimensionless wind speed ratio *R*_w_ is given by Eq. (22).

22 $$R_{w} = V_{p} /V_{S1} ,$$

where *V*_*p*_ and *V*_*S*1_ are the experimental wind velocity of measurement point and the Meteorological Station S1, respectively. Then, the actual ambient wind velocity of each measurement point could be calculated according to the actual wind speed measured by the Meteorological Station S1 and the relative magnitude of wind speed between the measurement points and the Meteorological Station S1.

**Figure 12:**
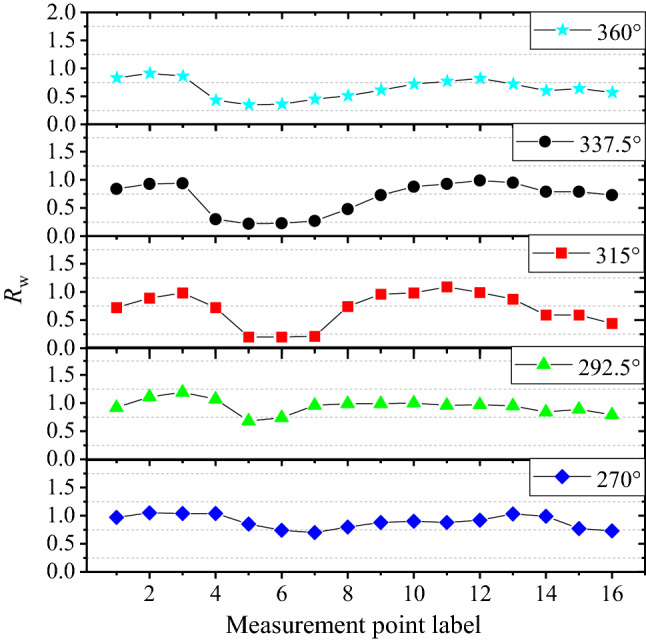
Variation of *R*_w_ with different measurement points.

Figure 12 illustrates the variation of *R*_w_ with different measurement points. When the directions of ambient wind were 270° and 292.5°, the wind speeds at A1–A4 and A10–A14 were basically the same as those of the Meteorological Station S1. Moreover, the measurement points A5–A9 were located on the north side of the ridge, which blocked the airflow movement and reduced the wind speed. Therefore, the wind speeds at these points were lower than those of the Meteorological Station S1. The wind speeds of A15 and A16 were significantly lower than those of S1. When the directions of ambient wind were 315°, 337.5°, and 360°, the wind speeds of all measurement points were generally lower than those of the Meteorological Station S1. Furthermore, the wind speeds of A4–A8 were greatly reduced due to the obvious windbreak effect of the ridge.

**Figure 13:**
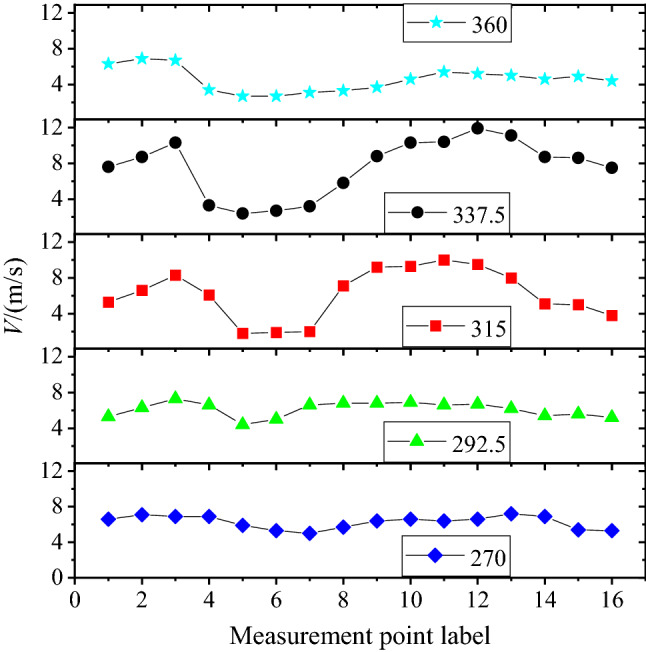
Variation of velocity with different measurement points.

The ambient wind velocity and the direction of the measurement point in the gliding slope are calculated and presented in Table 4. The variation of ambient wind velocity with different measurement points is depicted in Fig. 13. It can be seen that the ambient wind velocity was generally higher due to the higher altitude at the start of the alpine downhill track. Additionally, at the position near the end of the track, the wind speed was relatively small due to the relatively low altitude. The ambient wind velocity between A8–A13 sections of the track was the highest in the entire track. In particular, when the directions of ambient wind were 315° and 337.5°, the ambient wind velocity could be over 10 m/s, which had a significant impact on the competition and needed to be paid more attention to.

**Table 4:** Statistics of ambient wind velocity and direction in the gliding slope.

Measurement point	270°/W		292.5°/WNW		315°/NW		337.5°/NNW		360°/N	
	Direction (°)	Velocity (m/s)	Direction (°)	Velocity (m/s)	Direction (°)	Velocity (m/s)	Direction (°)	Velocity (m/s)	Direction (°)	Velocity (m/s)
A1	271	6.6	290	5.3	319	5.3	344	7.6	358	6.3
A2	271	7.1	292	6.3	323	6.6	346	8.7	359	6.9
A3	276	6.9	297	7.3	325	8.3	345	10.3	356	6.7
A4	277	6.9	295	6.6	320	6.1	340	3.3	353	3.4
A5	281	5.9	292	4.4	311	1.8	328	2.4	342	2.7
A6	282	5.3	296	5.0	315	1.9	329	2.7	337	2.7
A7	286	5.0	296	6.6	316	2.0	331	3.2	339	3.1
A8	279	5.7	294	6.8	316	7.1	332	5.8	342	3.3
A9	275	6.4	291	6.8	314	9.2	332	8.8	343	3.7
A10	274	6.6	291	6.9	315	9.3	333	10.3	346	4.6
A11	273	6.4	292	6.6	316	10.0	334	10.4	349	5.4
A12	272	6.6	296	6.7	323	9.5	339	11.9	350	5.2
A13	267	7.2	294	6.2	326	8.0	344	11.1	356	5.0
A14	264	6.9	280	5.4	325	5.1	346	8.7	359	4.6
A15	263	5.4	287	5.6	322	5.0	346	8.6	358	4.9
A16	266	5.3	283	5.2	311	3.8	340	7.5	358	4.4

### Evaluation of the effects of ambient wind

The effect of ambient wind on the performance of the skier is a superimposition of that on each section when completing the entire competition. Based on the kinematic model developed in "Methodology", gliding time was calculated for the five ambient wind directions with the highest wind frequency as well as the case of no wind. In the present study, the parameters setup for the simulation were: *m* = 72 kg, *μ* = 0.02^34,35^, and *ρ* = 1.25 × 10^3^ kg/m^3^. Based on the results presented in "Estimation of the drag area CdA" and to simplify the calculations, and considering that the average speed of alpine downhill exceeds 25 m/s, the values for *C*_*d*_ and *A* were 0.5 and 0.4 m^2^, respectively, which resulted in a constant value of *C*_*d*_*A* (0.2 m^2^). In addition, the initial kinematic velocity *v*_0_ and time consumed *t*_0_ were set to 10 m/s and 2 s, respectively.

**Figure 14:**
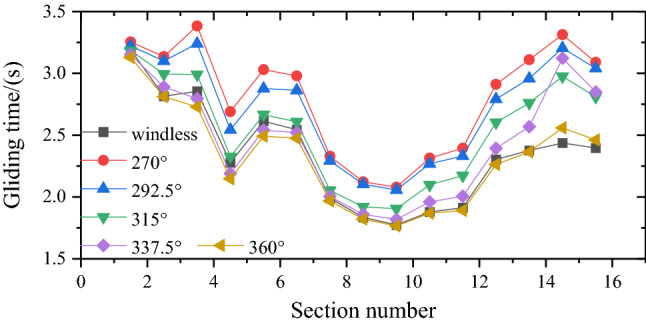
Gliding time of each section under the action of ambient wind.

Figure 14 illustrates the gliding time of each section under the action of ambient wind. The results for windless condition are also shown in Fig. 14. The impact of different directions of ambient wind on the gliding time of downhill skier to complete the whole race was different. When the direction of ambient wind was 360°, the gliding time of most sections was lower than that in the case of no wind. When the directions of ambient wind were 315° and 337.5°, in the front and middle sections of the entire track, the gliding time was basically the same as the no wind case. On the other hand, at the end of the track, the ambient wind had a great negative impact on the gliding time. When the directions of ambient wind were 270° and 392.5°, the gliding time of all sections was higher than that in the case of no wind, indicating that the negative impact of ambient wind in these directions on the performance of downhill skier needed more attention.

Table 5 presents the results of finish time at different ambient wind directions during the key sections of alpine downhill track. The results corresponding to the case of no wind are also presented in Table 5 for the purpose of comparison. It is obvious that the most unfavorable ambient wind direction for skiing performance was 270°, which can increase the time by up to 19.75% as compared to the case of no wind. When the ambient wind direction was 360°, the finish time can be reduced by 1.29%.

**Table 5:** Statistics of finish time under different ambient wind directions.

Conditions	Windless	270°/W	292.5°/WNW	315°/NW	337.5°/NNW	360°/N
Time (s)	35.20	42.15	40.90	38.07	36.67	34.75
*∆*t	0	6.95	5.70	2.87	1.47	− 0.45
Difference (%)	0	19.75	16.19	8.15	4.19	− 1.29

## Conclusions

The present investigation provides a combined method of field measurements, wind tunnel tests and kinematic modelling to evaluate the effect of ambient wind on the performance of alpine downhill skiers. Considering the effect of ambient wind, a kinematic model of the alpine downhill skier-ski system was established through some reasonable simplifications, followed by the derivation of equations of motion for straight gliding and turning. In addition, the CNASC downhill track was taken as an example to describe the evaluation process in detail. The racing finish time for the key section of the CNASC downhill track was calculated using time iteration.

As for the CNASC downhill track, from the field measurements and scale terrain model wind tunnel test, five critical wind directions of 270°, 292.5°, 315°, 337.5° and 360° were identified. When the wind direction was 337.5°, the maximum median wind speed can be up to 12 m/s. The results calculated by the modelling simulation show that, compared with not considering the ambient wind, for the 270° ambient wind direction, the finish time would increase by 19.75%, whereas for the 360° ambient wind direction, the ambient wind was beneficial to the racer’s performance. Meanwhile, the finish time was shortened by 1.29%.

The present investigation developed a combined evaluation method and revealed some interesting results about the effect of the ambient wind on the gliding time of alpine downhill skier, which can provide guidance for athletes or coaches for training purposes and developing suitable coping strategies. One limitation of this study was that the established model did not take the ski-snow interaction into consideration, which results in an approximation of the calculated friction. Additionally, the difference in trajectory between the COM and skis was not focused on, which may have a significant impact on the further works, such as the optimization of trajectory.

## Data Availability

The datasets generated during and/or analyzed during the current study are available from the corresponding author on reasonable request.
